# Dimethyl Itaconate Attenuates CFA-Induced Inflammatory Pain *via* the NLRP3/ IL-1β Signaling Pathway

**DOI:** 10.3389/fphar.2022.938979

**Published:** 2022-07-22

**Authors:** Jiaqi Lin, Jinxuan Ren, Bin Zhu, Yi Dai, Dave Schwinn Gao, Suyun Xia, Zhenzhen Cheng, Yangyuxin Huang, Lina Yu

**Affiliations:** Department of Anesthesiology, Second Affiliated Hospital of Zhejiang University School of Medicine, Hangzhou, China

**Keywords:** dimethyl itaconate, inflammatory pain, macrophages, microglia, NLRP3 inflammasome complex, IL-1β

## Abstract

Itaconate plays a prominent role in anti-inflammatory effects and has gradually been ushered as a promising drug candidate for treating inflammatory diseases. However, its significance and underlying mechanism for inflammatory pain remain unexplored. In the current study, we investigated the effects and mechanisms of Dimethyl Itaconate (DI, a derivative of itaconate) on Complete Freund’s adjuvant (CFA)-induced inflammatory pain in a rodent model. Here, we demonstrated that DI significantly reduced mechanical allodynia and thermal hyperalgesia. The DI-attenuated neuroinflammation was evident with the amelioration of infiltrative macrophages in peripheral sites of the hind paw and the dorsal root ganglion. Concurrently, DI hindered the central microglia activation in the spinal cord. Mechanistically, DI inhibited the expression of pro-inflammatory factors interleukin (IL)-1β and tumor necrosis factor alpha (TNF-α) and upregulated anti-inflammatory factor IL-10. The analgesic mechanism of DI was related to the downregulation of the nod-like receptor protein 3 (NLRP3) inflammasome complex and IL-1β secretion. This study suggested possible novel evidence for prospective itaconate utilization in the management of inflammatory pain.

## Introduction

Chronic inflammatory pain is associated with a number of clinical diseases such as osteoarthritis, rheumatoid arthritis, and fibromyalgia (Tracey et al., 2019). Long-term inflammatory pain causes deterioration in the patients’ health-related quality of life and even increases depressive symptoms and suicidal tendencies (Gaskin and Richard, 2012; Reid et al., 2015). Inflammatory pain originates from chemical stimuli, tissue damage, or autoimmune activation that causes nociceptors to respond to various stimuli and elicits pain hypersensitivity. Peripheral stimulation directly promotes the local release of inflammatory mediators, including prostaglandins, histamine, and neurogenic factors, which subsequently trigger a series of chain reactions, including neurogenic inflammation and peripheral and central sensitization, and eventually manifest as pain (Kamaldin et al., 2013; Gupta and Harvima, 2018). At present, non-steroidal anti-inflammatory drugs (NSAIDs) are recommended as the first-line pharmacotherapy to relieve pain and inflammation (Bannister et al., 2017). However, the long-term gastrointestinal side effects of these drugs greatly limit their clinical application. Thus, there is an urgent need to find new, safe, and effective anti-inflammatory pain medications.

More evidence shows that the accumulation, proliferation, and activation of peripheral macrophages and central glial cells play key roles in peripheral and central pain sensitization (Saika et al., 2019; Staats Pires et al., 2020). Activation of macrophages in DRG has been observed in many other pain models and correlates with the onset of pain genesis (Xie et al., 2006). Macrophages produce various inflammatory mediators, such as inflammatory cytokines, growth factors, and lipids after tissue injury and infection, directly leading to primary hyperalgesia at sites of injury or inflammation (Le Bars et al., 2001; Willemen et al., 2014). Microglial cells, the resident immune cells in the central nervous system, exhibit many immunological characteristics with peripheral macrophages (Hanisch and Kettenmann, 2007). Ever-increasing studies have found that many types of inflammatory mediators derived from activated microglia actively participate in the occurrence and development of pain (Ji and Suter, 2007; Chen et al., 2018). Furthermore, microglial and microglia-derived pro-inflammatory cytokines were inhibited by local microinjection of the microglial inhibitor minocycline, or neutralizing antibodies could help control and manage acute painful behaviors, more importantly, their transition to persistent hypersensitive pain conditions (Raghavendra et al., 2003; Grace et al., 2016).

The typical pro-inflammatory cytokine interleukin (IL)-1β has been shown to be produced by peripheral macrophages and activated microglia in the dorsal horn and is known to be upregulated in the various acute and chronic pain disorders (Binshtok et al., 2008; Gustafson-Vickers et al., 2008; Takeda et al., 2008; Ren and Torres, 2009). Intrathecal injection of IL-1β has a profound and long-lasting effect on pain hypersensitivity (Ren and Torres, 2009). IL-1β was initially produced as a cytosolic preprotein, requiring proteolysis at specific sites to activate its biological functions and release it outside the cell. The cleavage and subsequent secretion of IL-1β are mediated by the nod-like receptor protein 3 (NLRP3) inflammasome (Davis et al., 2011; Chan and Schroder, 2020). After activation of NLRP3, it couples with apoptosis-associated speck-like protein (ASC) and Caspase-1, thus forming NLRP3 inflammasome, thereby regulating pro-IL-1β cleaved into a mature form and quickly released into the extracellular environment to cause inflammation and pain (Cullen et al., 2015). Therefore, inhibiting the inflammasome complex and reducing the release of IL-1β are particularly important for the treatment of pain.

Itaconate is an α, β-unsaturated dicarboxylic acid (C5H6O4), an essential intermediate metabolite isolated from the tricarboxylic acid cycle in immune cells, especially macrophages. It is derived from cis-aconitate decarboxylation mediated by immune response gene one in the mitochondrial matrix (Lin et al., 2021). In recent years, the anti-inflammatory effect of itaconate has gradually been discovered and has attracted significant attention (Bambouskova et al., 2018; Mills et al., 2018). It can activate nuclear factor E2-related factor 2 (Nrf2) by alkylating kelch-like ECH-associated protein 1 (Keap1) to initiate anti-inflammatory and antioxidant responses (Mills et al., 2018). Itaconate could also target activating transcription factor 3 (ATF3)- an inhibitor of κB-ζ (IκBζ) pathway to mediate the inflammatory response (Bambouskova et al., 2018). Recent studies have also found that itaconate can dissociate NLRP3 from NEK7 through alkylation, thereby inhibiting the formation of inflammasome complexes and reducing the inflammatory progression of crystal-stimulated peritoneal inflammation (Hooftman et al., 2020). Itaconate has emerged as a critical determinant and participated in the development and progression of inflammation and immunity. Currently, it is not yet known whether itaconate has an analgesic effect on inflammation-induced pain. This study explored itaconate’s ability to regulate the progression of CFA-induced inflammation in the peripheral and central nervous systems. We reported that dimethyl itaconate (DI), a derivative of itaconate, alleviated CFA-induced pain in mice. In addition, we found that DI inhibited the activation of peripheral macrophages and central microglia, which in turn reduced the progression of peripheral and central nervous inflammation. The possible mechanism was to inhibit the formation of the NLRP3 complex and reduced the release of IL-1β.

## Materials and Methods

### Animals

Adult (6–8 weeks) C57BL/6J mice weighing 20–25 g were obtained from SLAC Laboratory Animal Centre (Shanghai, CN), housed in a specific pathogen-free facility at 22°C and relative humidity of 30% under a constant 12-h day and night cycle, in the Second Affiliated Hospital of Zhejiang University, School of Medicine. Procedures conducted in this study were approved by the Zhejiang Animal Care and Use Committee and the Second Affiliated Hospital, School of Medicine, Zhejiang University (Batch number:2022088). All experiments were carried out with investigators blinded to viral content or drug treatment during behavioral testing.

### Induction of Paw Inflammation and Drug Treatment

Inflammation was induced using 20 μl of CFA (Sigma) administered subcutaneously into the left hind paw. As an internal control, the mice were treated with the same volume of sterile saline injections. From the second day, DI (Sigma, 10 mg/d, 20 mg/d) or Phosphate Buffered Saline (PBS) was injected intraperitoneally every day. Behavioral measurements of hyperalgesia were assessed on the same animals before and on 2, 4, 6 h and days 1, 2, 4, 6, 8, and 12 following CFA injection. For the experiment of itaconate pretreatment, 3 days after DI (20 mg/d) was injected intraperitoneally, and the CFA model was established on day 4.

### Behavioral Tests

Paw withdrawal frequencies (PWF) were defined as mechanical pain tests using nylon von Frey filaments (DanMic Global. Campbell, CA, United States) as a response to physical stimulation. In short, rodents were put on top of a Cartesian mesh plane in a Plexiglas chamber individually for 30 min of acclimation. A total of 0.07 and 0.4 g von Frey filaments were prodded perpendicularly with sufficient force to stimulate the plantar surface of both hind paws for 1–2 s and with a repeated simulation of 10 times. Paw withdrawal responses were scored as a percent response frequency, PWF ((number of paw withdrawals/10 trials) × 100 = % response frequency).

The thermal nociception stimulus for paw withdrawal latencies (PWL) was measured using a Model 336a plantar algesia device (IITC Inc. Life Science Instruments, Woodland Hills, CA, United States). Briefly, the mice were put on a glass plate in a Plexiglas chamber individually for 30 min of acclimation. The withdrawal latency is defined as the period between light striking the hind paw’s plantar surface and the paw flick or withdrawal. A maximum stimulation period of 20 s was set to avoid skin injury during the procedure. The mean recorded value between three measurements with 5-min intervals was used as PWL.

On day 6 of CFA administration (the fifth day after DI treatment), the rodent was sedated with 1% pentobarbital and transcardially perfused with 37°C normal saline and 4% PFA (pH, 7.4; 4°C) in 0.01 M PBS. Then, the subcutaneous tissue at inflammatory sites medial to the left hind paw was immediately removed and fixed in 4% paraformaldehyde. Tissues were then dehydrated, embedded in paraffin, and sliced with a thickness of 5 μm. Hematoxylin and Eosin (HE) were used to stain all slices.

### Immunohistochemistry

The slides were subsequently deparaffinized and incubated in 3% H2O2 (10 min) washed in PBS (5 min, 3 times), and 5% bovine serum albumin (BSA) was used for half an hour incubation period at 25°C. The sections were stained with primary antibodies, myeloperoxidase (MPO) (1:100, Santa Cruz), CD45 (1:200, Abcam), CD68 (1:200, Proteintech), inducible nitric oxide synthase (iNOS) (1:200, Abcam), arginase-1 (Arg1) (1:200, GeneTex) and incubated at 4°C overnight. The sections were washed in PBS (5 min, 3 times) and incubated with polymerized horseradish peroxidase-labeled goat anti-rabbit IgG (Wuhan Boster Company) at 37°C for 30 min and washed in PBS (5 min, 3 times). Sections were detected by DAB (Vector Labs) under the lens and counterstained with hematoxylin. Appropriate positive and negative controls were added for every immunostaining. Sections were dehydrated by graded and finally mounted by neutral gum. The staining intensity was evaluated as 0, no staining; 1, weak; 2, moderate; or 3, strong. In a double score system for the fluorescent intensity, the proportion of the positive cells was utilized to evaluate the reactiveness and multiplied to generate a score (IHC score range 0–300) as described.

Immunofluorescence was performed with frozen sections (L4-6 spinal cord, DRG) or paraffin sections (hind paw). Slices were washed in 10 mM PBS (5 min, 3 times) and a one-hour incubation with 5% BSA at room temperature. The following antibodies were added and left overnight (4°C): rabbit anti-ASC (1:200, CST), mouse anti-ASC (1:100, Santa Cruz), mouse anti-Caspase 1 (1:100, Santa Cruz), mouse anti-IL-1b (1:100, Santa Cruz), mouse anti-CD68 (1:200, Abcam), rabbit anti-CD206 (1:200, Abcam), rabbit anti-CD86 (1:200, Abcam), rabbit anti- Iba1 (1:200; Wako), F4/80 (1:200, Invitrogen). Subsequently, the slices were rinsed in 10 mM PBS (5 min, 3 times) and were incubated with corresponding second antibodies for 1 h at room temperature. Finally, the slices were mounted with diamidino-phenyl-indole (DAPI, Abcam) to stain for the nucleus. The slides were inspected under fluorescence with Leica DMI4000.

### Real-Time Polymerase Chain Reaction

The subcutaneous tissue medial to the plantar, DRG, and spinal cord tissue was used for total RNA isolation with TRIzol reagent (Invitrogen). The cDNA was synthesized from total RNA (1 μg) a Hifair II 1st Strand cDNA Synthesis SuperMix for qPCR (gDNA digester plus) (Yeasen, Shanghai). All primers’ sequences for qPCR listed in Table 1 were synthesized in Sango Biotech (Shanghai, CN). qPCR was carried out as follows: initial 2 min at 95°C followed by 40 cycles of 15 s at 95°C and 60 s at 60°C. qPCR was conducted according to the instructions using the SYBR-Green System (Yeasen, Shanghai). Relative gene expression was calculated using the 2^−ΔΔCt^ method, with β-Actin or tubulin as the internal control and reference gene.

**TABLE 1:** Specific primers used for qPCR.

	Forward primers	Reverse primers
β-Actin	AGG​CAT​TGT​GAT​GGA​CTC​CG	AGC​TCA​GTA​ACA​GTC​CGC​CTA
Tubulin	GAT​GCT​GCC​AAT​AAC​TAT​GCT​C	TTG​GAC​TTC​TTT​CCG​TAA​TCC​A
IL-1β	TCG​CAG​CAG​CAC​ATC​AAC​AAG​AG	AGG​TCC​ACG​GGA​AAG​ACA​CAG​G
TNF-α	ATG​TCT​CAG​CCT​CTT​CTC​ATT​C	GCT​TGT​CAC​TCG​AAT​TTT​GAG​A
IL-10	GCC​TGC​TCT​TAC​TGA​CTG​GC	AGC​TCT​AGG​AGC​ATG​TGG​CT

### Western Blotting

The L4-6 spinal cord, DRG, and hind paw protein were isolated on the 6th day after CFA injection (the fifth day after DI treatment). The tissues were lysed in Radioimmunoprecipitation Assay buffer (RIPA) which contained 0.05 M Tris (pH 7.4), 0.15 M sodium chloride, 1% sodium deoxycholate, 1% Triton X-100%, 0.1% sodium dodecyl sulphate. The supernatants were gathered after being centrifuged for 15 min at 4°C with 1500 RCF and the concentration of protein was measured using a bicinchoninic acid (BCA) assay kit (Beyotime, CN). The samples were boiled at 100°C for 5 min and transferred on a 10% sodium deoxycholate-polyacrylamide gel (Genshare Biology, CN) for electrophoretic separation and immunoblotting before being transferred electrophoretically with current of 0.25 A onto a PVDF film (Millipore, Burlington, MA, United States) for 90 min. The films were blocked with 5% nonfat milk TBST for 1 h and then incubated at 4°C overnight with the following antibodies: mouse anti-GAPDH (1:1,000, Zhongshan Golden Bridge Biotechnology), rabbit anti-ASC antibody (1:1,000, CST), rabbit anti-NLRP3 antibody (1:1,000, CST), mouse anti-Caspase one antibody (1:200, Santa Cruz), mouse anti-ASC antibody(1:200, Santa Cruz), mouse anti-IL-1b antibody (1:200, Santa Cruz). The protein was detected by HRP-conjugated anti-mouse or rabbit secondary antibody (1:5,000, Jackson ImmunoResearch). Clarity Western ECL Substrate (EMD Millipore) was utilized for visualization and ChemiDoc XRS with System (Bio-Rad) for exposure. The western blot intensity was determined using Fiji ImageJ.

### Statistical Analyses

All of the results used the notation of mean ± SEM. Two-way ANOVA followed by Tukey’s posthoc test was used for withdrawal thresholds and the thermal latency between groups. One-way ANOVA with Tukey’s procedure was utilized to evaluate the biochemical results (GraphPad Prism 8). Significance between different groups was set at *p* < 0.05.

## Results

### DI Reduces Inflammatory Pain Development

**FIGURE 1:**
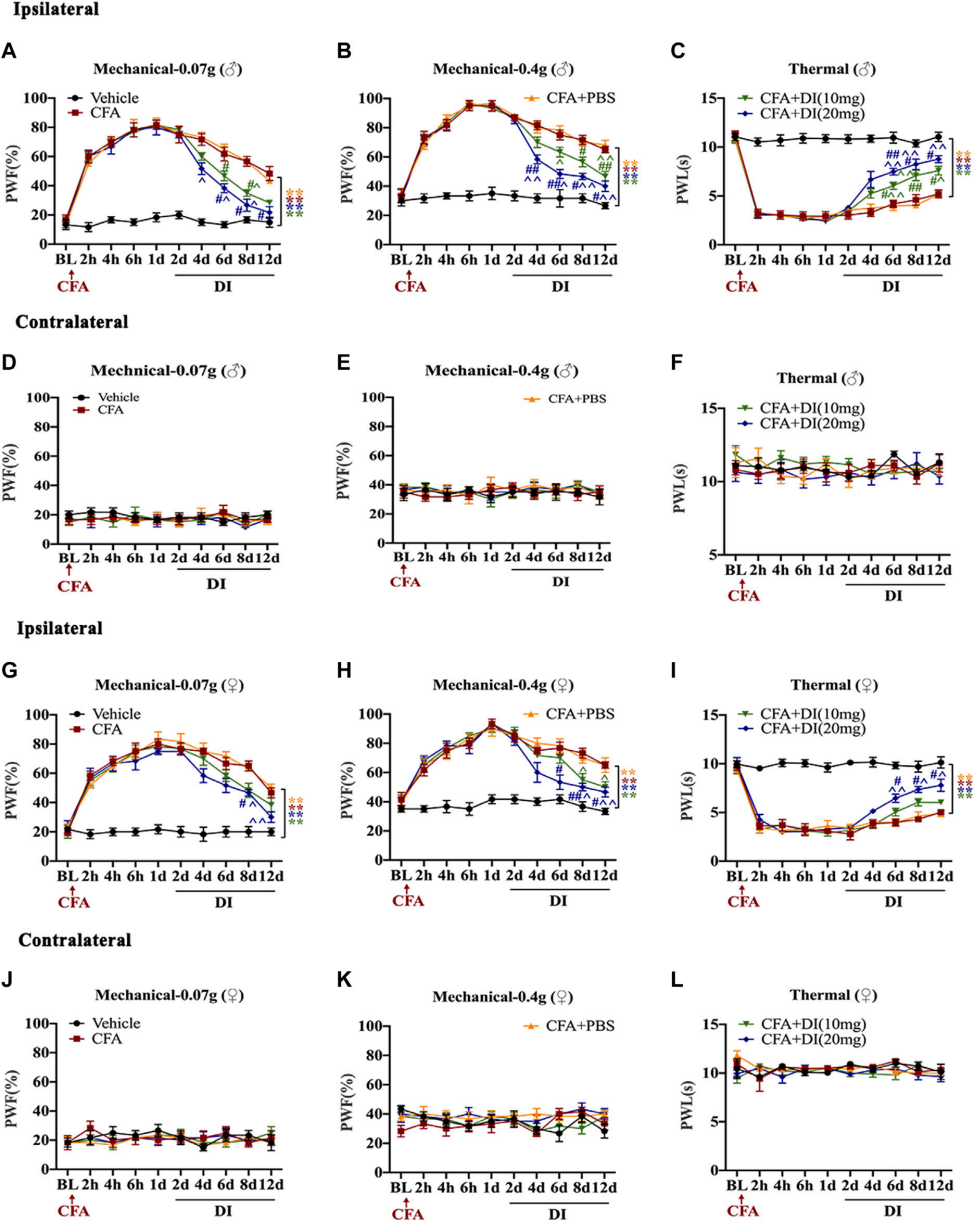
Effects of DI on CFA-induced mice inflammatory pain model. **(A,B)** The effect on mechanical allodynia (0.07 or 0.4 g) of the ipsilateral hind paw in male mice. F_group_ (36, 200) = 9.377 for **(A)**, F_group_ (36, 200) = 9.944 for **(B)**. **(C)** The effect on thermal hyperalgesia ipsilateral hind paw in male mice. F_group_ (36, 200) = 13.93. **(D–F)** The effect of mechanical allodynia and hyperalgesia of the contralateral hind paw. F_group_ (36, 200) = 0.3595 for **(D)**, F_group_ (36, 200) = 0.3423 for **(E)**, F_group_ (36, 200) = 0.5646 for **(F)**. **(G–H)** The effect on mechanical allodynia (0.07 or 0.4 g) of the ipsilateral hind paw in female mice. F_group_ (36, 200) = 5.347 for **(G)**, F_group_ (36, 200) = 4.478 for **(H)**. **(I)** The effect on thermal hyperalgesia ipsilateral hind paw in female mice. F_group_ (36, 200) = 6.262. **(J–L)** The effect of mechanical allodynia and hyperalgesia of the contralateral hind paw. F_group_ (36, 200) = 0.4229 for **(J)**, F_group_ (36, 200) = 0.9263 for **(K)**, F_group_ (36, 200) = 1.391 for **(L)**. Data values are expressed as mean ± SEM. N = 6 mice/group. ^**^ *p* < 0.01 versus the vehicle group; ^#^ *p* < 0.05, ^##^ *p* < 0.01 versus the CFA group; ˄*p* < 0.05, ˄˄*p* < 0.01 versus the CFA + PBS group.

To investigate the role of DI in chronic inflammatory pain, the left hind paw was infused with CFA. PWF to 0.07 and 0.4 g von Frey filament stimuli responded with robust increases 2 h after injection. The ipsilateral PWL to thermal nociception changed with rapid reductions in 2 h and subsequently reached a steady state on day 1 (Figures 1A–C). Daily intraperitoneal injection of DI started on day 2. DI (20 mg) attenuated CFA-induced mechanical allodynia as demonstrated by the reduction of PWF to mechanical stimuli from day 4 and improved CFA-induced thermal hyperalgesia as indicated by the increase in PWL to heat stimulation from day 6 to day 12 compared to the CFA and CFA + PBS groups (Figures 1A–C). The low dose of DI (10 mg) began to relieve the mechanical and thermal hyperalgesia in mice on the 6th-day post CFA injection. Due to the gender difference in pain, we also verified the relief of DI on inflammatory pain in female mice. Same as male mice, DI can relieve mechanical and thermal hyperalgesia. DI (20 mg) relieves mechanical pain on the 8th day and thermal hyperalgesia on the 6th day after CFA injection (Figures 1G–I). The low dose of DI only relieves mechanical pain on the 8th day, but there is no significant difference in thermal hyperalgesia. As expected, there was no noticeable change in the PWT or PWF of the contralateral plantar (Figures 1D–F,J–L). These results suggested that DI could attenuate pain behavior.

### DI Pretreatment Did Not Alleviate Inflammatory Pain Development

**FIGURE 2:**
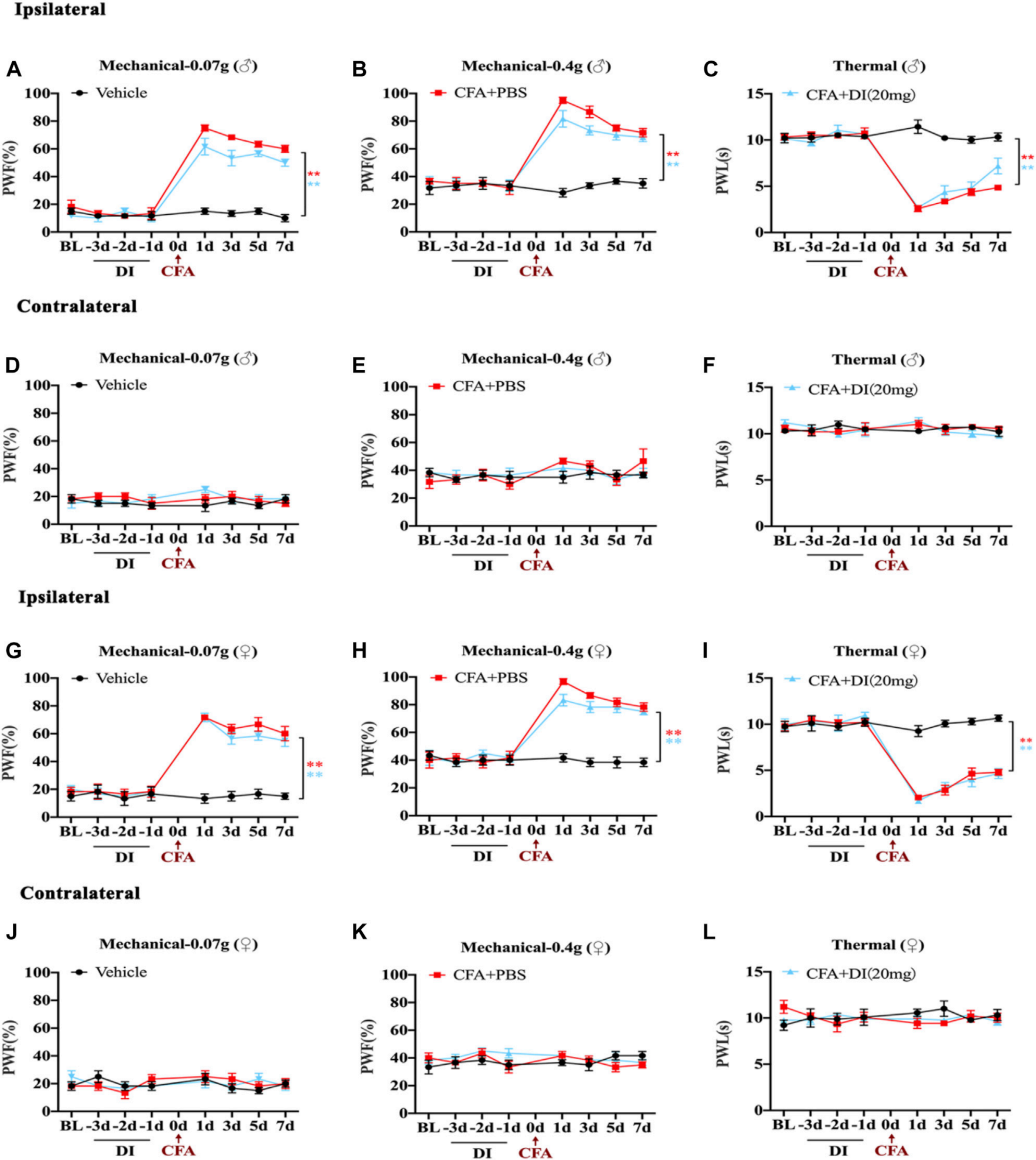
Effects of DI pretreatment on CFA-induced mice inflammatory pain model. **(A,B)** The effect on mechanical allodynia (0.07 or 0.4 g) of the ipsilateral hind paw in male mice. F_group_ (14, 80) = 26.60 for **(A)**, F_group_ (14, 80) = 15.17 for **(B)**. **(C)** The effect on thermal hyperalgesia ipsilateral hind paw in male mice. F_group_ (14, 80) = 24.28 for **(C)**. **(D–F)** The effect of mechanical allodynia and hyperalgesia of the contralateral hind paw. F_group_ (14, 80) = 0.8967 for **(D)**, F_group_ (14, 80) = 0.8594 for **(E)**, F_group_ (14, 80) = 1.560 for **(F)**. **(G,H)** The effect on mechanical allodynia (0.07 or 0.4 g) of the ipsilateral hind paw in female mice. F_group_ (14, 80) = 16.72 for **(G)**, F_group_ (14, 80) = 12.55 for **(H)**. **(I)** The effect on thermal hyperalgesia ipsilateral hind paw in female mice. F_group_ (14, 80) = 17.73 for **(I)**. **(J–L)** The effect of mechanical allodynia and hyperalgesia of the contralateral hind paw. F_group_ (14, 80) = 0.9991 for **(J)**, F_group_ (14, 80) = 1.161 for **(K)**, F_group_ (14, 80) = 1.198 for **(L)**. Data values are expressed as mean ± SEM. N = 6 mice/group. **p < 0.01 versus the vehicle group.

To study the role of DI pretreatment in chronic inflammatory pain, DI (20 mg) was pre-administered for 3 days in naive mice, and CFA was injected into the left hind paw. As shown in the figure, DI (20 mg) pretreatment did not affect baseline thermal pain thresholds and mechanical pain thresholds in naive mice (Figures 2A–C,G–I). PWF to 0.07 and 0.4 g von Frey filament stimuli responded with robust increases, and ipsilateral PWL to thermal nociception changed with rapid reductions on day 1. DI (20 mg) pretreatment did not alter CFA-induced mechanical allodynia and thermal hyperalgesia compared to CFA + PBS groups (Figures 2A–C,G–I). The contralateral plantar has no noticeable change in the PWT or PWF (Figures 2D–F,J–L). These results suggested that DI pretreatment could not attenuate pain behavior.

### DI Treatment Antagonizes CFA-Induced Local Inflammation

**FIGURE 3:**
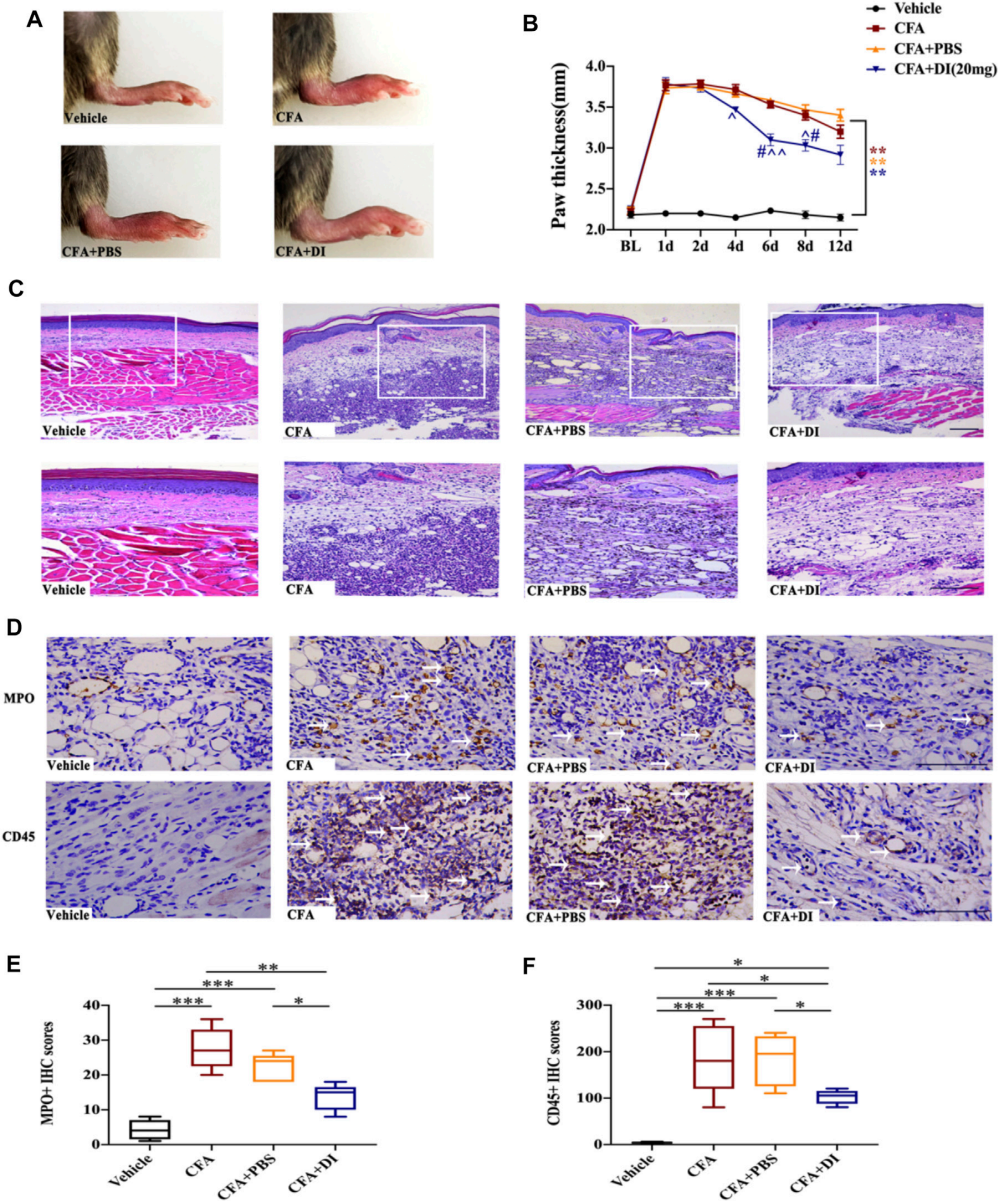
Effect of DI on paw thickness and Inflammatory cell infiltration in CFA model. **(A)** Macroscopic images of hind paw derived from vehicle, CFA and CFA + PBS and CFA + DI-treated mice. **(B)** Hind paw thickness (mm) as a function of time (days) was measured in each group. F_group_ (18, 105) = 22.88 for **(B)** (N = 6 mice/group). **(C)** HE staining. Inflammation with abundant lymphocytes and sparse neutrophilic granulocytes in a CFA mouse with hypodermic injection and low-grade inflammation in a CFA mouse with a hypodermic injection of DI (20 mg). This experiment was repeated independently 3 times and similar results were obtained. The scale bar represents 200 and 100 μm. **(D)** Photomicrographs representing MPO and CD45 immunoreactivity in each group. Arrowheads indicate positive cells. ^**^ *p* < 0.01 versus the vehicle group; ^#^ *p* < 0.05 versus the CFA group; ˄*p* < 0.05, ˄˄*p* < 0.01 versus the CFA + PBS group. **(E,F)** MPO score and CD45 score. F_group_ (3, 16) = 16.68 for **(E)**, F_group_ (3, 16) = 28.36 for **(F)**. The scale bar represents 50 μm. This experiment was repeated independently 3–5 times and similar results were obtained. ^*^ *p* < 0.05, ^**^ *p* < 0.01 and ^***^ *p* < 0.001.

Mice with inflammatory pain are usually accompanied by swelling of the painful site and infiltration of inflammatory cells. After CFA injection, the subcutaneous tissue at inflammatory sites medial to the left hind paw swelled rapidly, and the thickness of the hind paw increased from 2–2.5 to 3.5–4 mm (Figures 3A,B). Moreover, HE staining on the 6th-day post-CFA injection of the CFA and CFA + PBS hind paw samples displayed numerous inflammatory cells invading the nearby dermal samples (Figure 3C), and immunohistochemical analysis revealed a large number of MPO-positive neutrophils, CD45-positive immune cells (white arrows) infiltration. When injected with DI (20 mg), it significantly reduced hind paw swelling (Figures 3A,B) and inflammatory cell infiltration compared with CFA and CFA + PBS groups (Figures 3C–F, *p* < 0.05). The above data indicated that DI could inhibit local inflammation.

### DI Inhibits Macrophage Activation in the Hind Paw and DRG and Microglia Activation in the Spinal Cord

**FIGURE 4:**
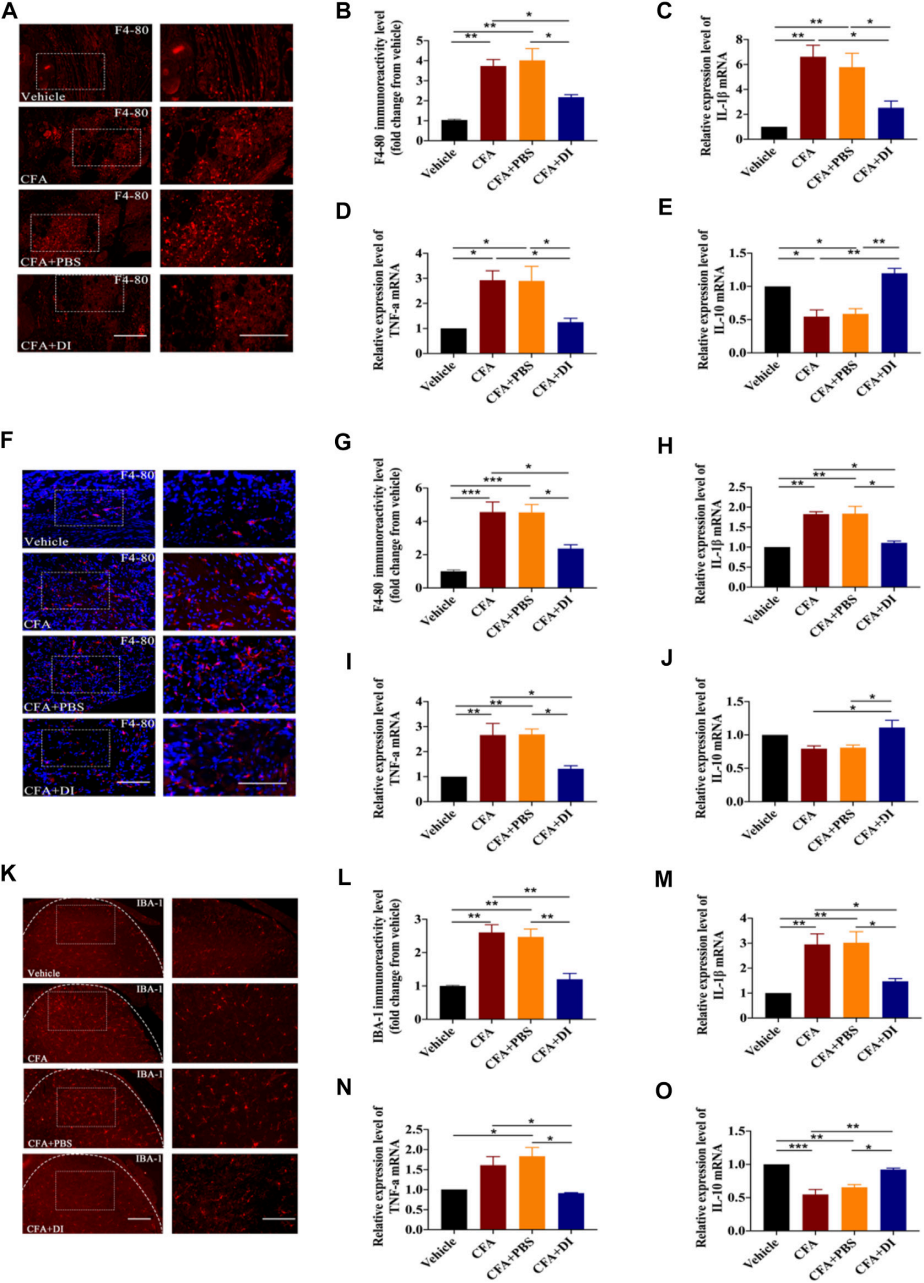
Effect of DI on macrophage and microglia activation. **(A)** Immunostaining of F4-80 (red) showing macrophages activation in paw tissue. The scale bar represents 100 μm (left) and 50 μm (right). **(B)** Representative immunofluorescence staining level for F4-80, fold change versus the vehicle group. F_group_ (3, 8) = 16.65 for **(B)**. Real-time PCR were used to detect pro-inflammatory factors IL-1β, F_group_ (3, 12) = 11.87 for **(C)**, TNF-α, F_group_ (3, 8) = 8.567 for **(D)** and anti-inflammatory factor IL-10, F_group_ (3, 8) = 18.75 for **(E)**. **(F)** Immunostaining of F4-80 (red) and DAPI (blue) showing macrophages activation in DRG. The scale bar represents 100 μm (left) and 50 μm (right). **(G)** Representative immunofluorescence staining level for F4-80, fold change versus the vehicle group. F_group_ (3, 12) = 18.83 for **(G)** .Real-time PCR were used to detect pro-inflammatory factors IL-1β, F_group_ (3, 12) = 20.84 for **(H)**, TNF-α, F_group_ (3, 12) = 11.49 for **(I)** and anti-inflammatory factor IL-10, F_group_ (3, 12) = 6.271 for **(J)**. **(K)** Immunostaining of IBA-1 (red) showing microglia activation in spinal cord. F_group_ (3, 8) = 19.96 for **(K)**. The scale bar represents 200 μm (left) and 100 μm (right). **(L)** Representative immunofluorescence staining level for IBA-1, fold change versus the vehicle group. Real-time PCR analysis of IL-1β, F_group_ (3, 8) = 10.92 for **(M)**, TNF-α, F_group_ (3, 8) = 8.719 for **(N)** and IL-10, F_group_ (3, 12) = 23.67 for **(O)** expression relative to vehicle levels in spinal cord. This experiment was repeated independently 3 times and similar results were obtained. ^*^ *p* < 0.05, ^**^ *p* < 0.01 and ^***^ *p* < 0.001.

The duality of peripheral macrophage and central microglia activations are the primary pathways responsible for inflammatory pain’s start point and progression (Sagar et al., 2011; Allen et al., 2020). We first examined whether DI administration affected macrophage activation in the ipsilateral hind paw with F4/80 marker immunofluorescence tagging. The numbers of F4-80^+^ macrophages exhibited profuse activation in the ipsilateral hind paw after CFA injection compared to those in the vehicle group (Figures 4A,B). However, CFA mice treated with DI (20 mg) showed markedly scarcer F4-80^+^ cells (*p* < 0.05). Treatment with DI also significantly decreased IL-1β, TNF-α mRNA expression relative to the CFA group (Figures 4C,D, *p* < 0.05) and promoted anti-inflammatory cytokine IL-10 (Figure 4E, *p* < 0.05). Similar results were also found in the DRG consistent with the hind paw. DI also suppressed macrophages activation in the ipsilateral DRG (Figures 4F,G, *p* < 0.05), and inhibited pro-inflammatory factors TNF-α, IL-1β (Figures 4H,I, *p* < 0.05), and reversed the reduction of anti-inflammatory factors IL-10 induced by CFA (Figure 4J, *p* < 0.05). Moreover, the numbers of Iba1 positive cells were significantly increased, and the expressions of these pro-inflammatory cytokines were also upregulated in CFA and CFA + PBS groups (Figures 4K,L, *p* < 0.05). DI intervention mitigated this expression, similar to that of the vehicle group (Figures 4M–O, *p* < 0.01). The above data show that DI may inhibit macrophage/microglia activation and the expression of pro-inflammatory factors and promote the activation of anti-inflammatory factors in peripheral and central systems.

### DI Reduced M1 Polarization and Facilitated M2 Polarization

**FIGURE 5:**
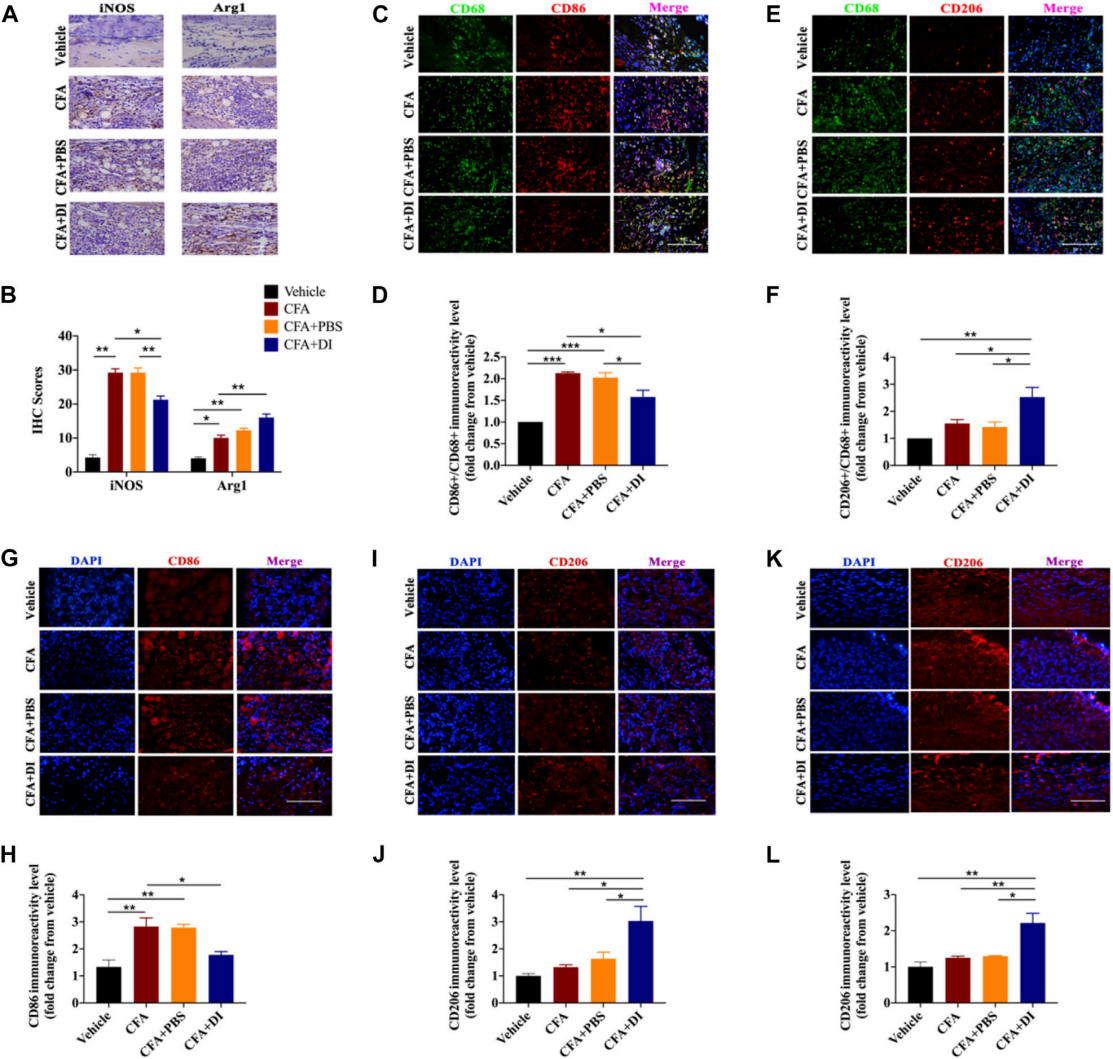
Effect of DI on macrophage/microglia polarization. **(A)** Photomicrographs representing iNOS and Arg1 immunoreactivity in each group. Arrowheads indicate positive cells. **(B)** iNOS score and Arg1 score were detected the same as above. F_group_ (3, 12) = 113.0 for iNOS. F_group_ (3, 12) = 42.18 for Arg1. The scale bar represents 50 μm. **(C,E)** Immunofluorescent staining for CD68 (Green), CD86 (red), and CD206 (red), and nuclei were counterstained using DAPI (blue). The scale bar represents 50 μm. Representative immunofluorescence staining level for CD86+/CD68+, F_group_ (3, 8) = 28.14 for **(D)** and CD206+/CD68+, F_group_ (3, 8) = 9.312 for **(F)**, fold change versus the vehicle group. Immunostaining of CD86 **(G)** and CD206 **(I)** in DRG. The scale bar represents 50 μm. Representative immunofluorescence staining level for CD86, F_group_ (3, 8) = 11.12 for **(H)** and CD206, F_group_ (3, 8) = 8.999 for **(J)**, fold change versus the vehicle group. **(K)** Immunostaining of CD206 in the spinal cord. The scale bar represents 50 μm. **(L)** Representative immunofluorescence staining level for CD206, F_group_ (3, 8) = 12.42, fold change versus the vehicle group. This experiment was repeated independently 3 times and similar results were obtained. ^*^ *p* < 0.05, ^**^ *p* < 0.01 and ^***^ *p* < 0.001.

Different subsets of macrophages in the injured hind paw were characterized. CFA triggered an increase of macrophages in the injured hind paw. Moreover, local inflammation was associated with increased iNOS, and Arg1 expression (Figures 5A,B). DI (20 mg) inhibited the expression of iNOS but further increased Arg1 expression compared with CFA and CFA + PBS group (*p* < 0.05). There was a reduction of M1 macrophages with marker CD86 (Figures 5C,D) and increased the proportion of M2 macrophages, and upregulated the expression of their marker CD206 (Figures 5E,F, *p* < 0.05). Similarly, there was a reduction of CD86 macrophages (Figures 5G,H) and an increase of CD206 macrophages in DRG treated with DI. (Figures 5I,J, *p* < 0.05). Similar results were also found in the spinal cord dorsal horn consistent with DRG. DI also reversed the reduction of CD206 positive cells induced by CFA. (Figures 5K,L), signifying DI might stimulate macrophage polarization from classically activated pro-inflammatory phenotypes to alternatively activated anti-inflammatory phenotypes.

### DI Inhibits the Activation of the NLRP3 Inflammatory Complex and Reduces the Release of IL-1β

**FIGURE 6:**
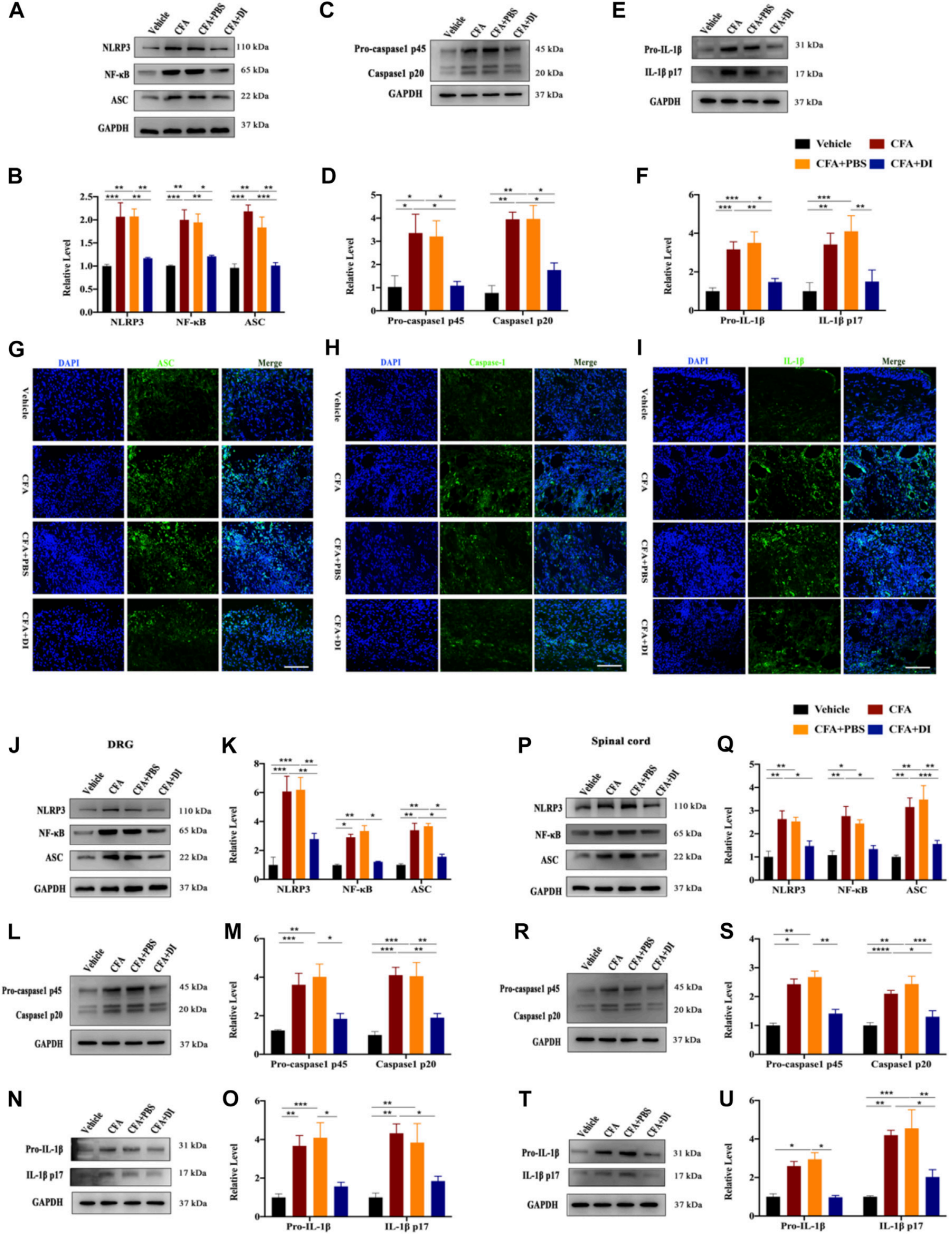
DI inhibited NLRP3 inflammasome activation and IL-1β secretion. **(A,B)** Western blotting bands and analysis of NLRP3, ASC, NF-κB in plantar tissue. F_group_ (3, 8) = 10.95 for NLRP3, F_group_ (3, 8) = 18.02 for ASC, F_group_ (3, 8) = 12.64 for NF-κB. **(C,D)** Western blotting bands and analysis of Caspase1 p45 and p20 in plantar tissue. F_group_ (3, 12) = 9.598 for Caspase1 p45, F_group_ (3, 12) = 16.47 for Caspase1 p20. **(E,F)** Western blotting bands and analysis of pro-IL-1β and IL-1β p17 in plantar tissue. F_group_ (3, 20) = 11.32 for pro-IL-1β, F_group_ (3, 20) = 7.736 for IL-1β p17. Immunofluorescent staining for ASC (green) **(G)**, Caspase-1 (green) **(H)**, IL-1β (green) **(I)**, the nuclei were stained blue with DAPI. The scale bar indicates 50 μm. **(J,K)** Western blotting bands and analysis of NLRP3, ASC, NF-κB in DRG. F_group_ (3, 16) = 11.44 for NLRP3, F_group_ (3, 16) = 23.45 for ASC, F_group_ (3, 16) = 29.99 for NF-κB. **(L,M)** Western blotting bands and analysis of Caspase1 p45 and p20 in DRG. F_group_ (3, 16) = 8.323 for Caspase1 p45, F_group_ (3, 16) = 12.98 for Caspase1 p20. **(N,O)** Western blotting bands and analysis of pro- IL-1β and IL-1β p17 in DRG. F_group_ (3, 16) = 9.598 for pro-IL-1β, F_group_ (3, 12) = 7.633 for IL-1β p17. **(P,Q)** Western blotting bands and analysis of NLRP3, ASC, NF-κB in spinal cord. F_group_ (3, 16) = 9.572 for NLRP3, F_group_ (3, 16) = 10.55 for ASC, F_group_ (3, 16) = 10.52 for NF-κB. **(R,S)** Western blotting bands and analysis of Caspase1 p45 and p20 in spinal cord. F_group_ (3, 12) = 17.36 for Caspase1 p45, F_group_ (3, 12) = 13.11 for Caspase1 p20. **(T,U)** Western blotting bands and analysis of pro- IL-1β and IL-1β p17 in spinal cord. F_group_ (3, 16) = 21.28 for pro-IL-1β, F_group_ (3, 16) = 10.23 for IL-1β p17. N = 3-5 biological repeats, 2 mice/group/repeat. ^*^ *p* < 0.05, ^**^ *p* < 0.01 and ^***^ *p* < 0.001.

The NLRP3-inflammasome complex is one of the most important regulators of inflammation. Expression of NLRP3, ASC, caspase-1, and IL-1β at the protein level of skin tissues were evaluated. The expressions of NLRP3 and ASC were higher in the CFA and CFA + PBS groups than in the vehicle group (Figures 6A,B). Following DI treatment, NLRP3 and ASC were significantly reduced (*p* < 0.05). We also tested the expression of NF-κB. DI inhibited the increase of NF-κB caused by CFA (Figures 6A,B). In addition, the caspase-1 p45 and IL-1β release were initiated by CFA injection. DI eliminated caspase-1 p20 and IL-1β p17 release compared with CFA and CFA + PBS groups (Figures 6C–F, *p* < 0.05). Immunofluorescence staining showed that positive ASC, IL-1β, and Caspase-1 in CFA and CFA + PBS groups were less than those in CFA and CFA + PBS groups (Figures 6G–I). We also detected the DI effect on the NLRP3 inflammatory complex in DRG. NLRP3 and NF-κB protein was found to increase, which was concomitant with high ASC level, while DI treatment reversed this activation (Figures 6J,K). We also observed an increase in the levels of caspase-1 p45 and p20 as well as the maturity and release of IL-1β compared to the vehicle group (Figures 6L–O). DI treatment reversed these results (*p* < 0.05). Consistent with plantar tissue and DRG results, DI reduced the formation of the NLRP3 complex and NF-κB in the spinal cord and inhibited the IL-1β activated into the section of IL-1β p17 (Figures 6P–U). In general, CFA significantly elevated the expression of the NLRP3 inflammasome, which was decreased by treatment with DI. These results are indicated that DI may alleviate inflammatory pain induced by CFA by inhibiting the NLRP3 inflammasome.

## Discussion

**FIGURE 7:**
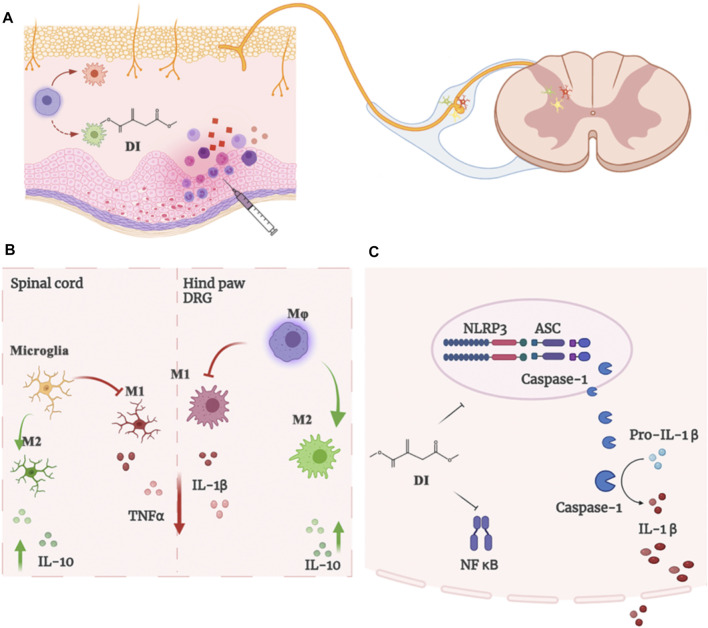
**(A)**The enhanced microglia proliferation in the spinal cord and the infiltration of macrophages into the DRG and paw tissue of the CFA mice contribute to the development of inflammatory pain. **(B)** DI regulated and inhibited the imbalance between pro-inflammatory and anti-inflammatory responses. **(C)** DI suppressed the assembly of NLRP3 inflammasome complexes and NF-κB, thereby inhibiting the secretion of IL-1β.

The overall goal of our study was to determine the analgesic effects of DI on inflammatory pain models. Our results found that the enhanced activation of macrophages into paw tissue and the DRG and microglia in the spinal cord contributes to the development of inflammatory pain. The analgesic mechanism of DI occurs via regulating the balance between pro-inflammatory and anti-inflammatory responses and suppressing the assembly of NLRP3 inflammasome complexes (Figure 7).

To our best knowledge, there were no reports describing the application of DI in inflammation pain. However, itaconate has been shown in previous studies to exert significant anti-inflammatory effects under various inflammatory conditions, including sepsis, neuroinflammation, bacterial or virus infection, rheumatoid arthritis, and pneumonia (Mills et al., 2018; Daly et al., 2020; Kuo et al., 2020; Lin et al., 2021). So we infer that the anti-inflammatory effect of itaconate has a therapeutic effect on inflammatory pain. In this study, we found that various DI (10 and 20 mg) doses could effectively reduce mechanical allodynia and thermal allodynia caused by CFA. Since the current research is based on the anti-inflammatory effects of itaconate to speculate its analgesic potential, other types of pain manifestations such as peripheral neuropathy, cancer pain, and acute pain still need to be further explored to broaden the application of itaconate. In addition, in view of the gender differences in pain, we also found that itaconate has analgesic effects in female mice. However, male mice displayed an immediate and significant reverse in PWF, whereas female mice that received the DI injection displayed a delayed analgesic effect. This may be due to gender differences in the prevalence and intensity of chronic inflammation (Glass et al., 2014). Recent studies have found that pretreatment with DI offered a protective effect on the macrophages later stimulated with LPS (Mills et al., 2018; Hooftman et al., 2020). We speculated DI pretreatment would prevent the development of CFA-induced inflammatory pain. However, contrary to the prediction, DI does not change the threshold of mechanical and thermal pain in naive mice and does not prevent the occurrence of inflammatory pain in mice. The possible reason is that DI does not change the threshold of mechanical and thermal pain in mice. Moreover, the short-term effect of rapid degradation or further metabolization of DI was not enough to release endogenous itaconate (Swain et al., 2020). It will quickly produce pharmacological effects in 3–4 h and is easily excreted from the body within 24 h (Wang et al., 1961), which may also explain its low toxicity. Taken together, 3 days of DI pre-administration was not adequate to reverse, mechanical pain, and thermal pain caused by CFA.

Meanwhile, we found that DI inhibited plantar skin swelling and inflammatory cell infiltration in plantar tissues. HE staining showed many inflammatory cells (MPO + neutrophils, CD45^+^ immune cells, macrophages) infiltrated around paw muscles. Long-term infiltration of a large number of inflammatory mediators will lead to unresolved inflammation (Ricardo et al., 2008; Murray and Wynn, 2011; Wynn et al., 2013). Among these inflammatory cells, macrophages occupy a large proportion. The F4/80 (encoded by Emr1), a mononuclear phagocyte marker, is solely expressed on the surface of macrophages and serves as a marker for mature macrophage tissues, including Langerhans cells in the skin, brain microglia, DRG macrophage, and Kupffer cells in the liver (Leenen et al., 1994). So F4-80 was chosen as a marker of DRG macrophage activation. Moreover, peripheral inflammatory processes lead to central sensitization of spinal cord circuits, which may lead to resultant hyperalgesia and allodynia (Pinho-Ribeiro et al., 2018; Yam et al., 2018). Our study found that DI treatment reduced macrophage over-activation in hind paw and DRG tissues. This may indicate that DI can further inhibit the occurrence of peripheral inflammation and prevent peripheral sensitization of pain. Microglia are resident macrophages in the central nervous system. Current studies indicate that the activation of microglia plays a critical role in the development of central sensitization in the spinal cord in the CFA model (Sagar et al., 2011; Liao et al., 2017). Minocycline (a non-selective microglial inhibitor) or propentofylline (a glia-modulating agent) can prevent or reverse mechanical and thermal hyperalgesia in rodents (Fernandez-Zafra et al., 2019). DI treatment also inhibited the activation of microglia in the spinal cord, indicating that itaconate can inhibit the formation of central sensitization. Macrophages usually differentiate into different functional phenotypes to regulate complex inflammatory processes. Inflammatory (M1) macrophages are characterized by high production of inflammatory cytokines and chemokines (IL-1β, TNFα, CCL3, CCL4, and iNOS), hence further intensifying inflammation (Liu et al., 2000; Mert et al., 2009; Nadeau et al., 2011; Sica and Mantovani, 2012). By contrast, M2 macrophages are characterized by expressing high levels of IL-10 and Arg1 to relieve the inflammatory condition (Sica and Mantovani, 2012). A recent study found that DI promoted the transition from M1 to M2, reducing the expression of inflammatory mediators in microglia treated with LPS and ATP (Yang et al., 2021). In our study, DI promoted Arg1 and CD206 and inhibited the expression of iNOS and CD86 in CFA-treated paw tissues. DI promoted the transformation of M2 macrophages to counterbalance the extensive inflammatory response and promote the resolution of inflammation. We also found that DI treatment could inhibit the activation of peripheral DRG macrophages, and excessive activation of DRG macrophages can cause peripheral sensitization. DI treatment also inhibited the expression of CD86 but promoted CD206 in DRG tissues and increased the expression of CD206 in the spinal dorsal horn. The regulation of anti-inflammatory and pro-inflammatory may be an important factor in the DI analgesic effect. Moreover, it was also found that DI can reduce the production of TNF-α and promote the expression of anti-inflammatory factors, IL-10. These cytokines help the recruitment of inflammatory cells to tissues in the periphery and central nervous system, thereby increasing the inflammatory process and pain (Das, 2010).

Studies have found that IL-1β produced by macrophages promotes the recruitment of neutrophils and lymphocytes at the inflammation site, eventually leading to an inflammatory cascade. On the other hand, mature IL-1β plays a vital role in initiating inflammatory pain hypersensitivity (Opree and Kress, 2000). IL-1β can also sensitize or directly activate nociceptors firing and glial activation to induce persistent pain (Safieh-Garabedian et al., 1995; Kawasaki et al., 2008; Pinho-Ribeiro et al., 2017). Blocking IL-1β with neutralizing antibodies can significantly alleviate inflammatory pain (Torres et al., 2009). In the CFA model, NLRP3 could lead to the activation of caspase-1 and elicited the maturation and secretion of the pro-inflammatory cytokines of IL-1β and IL-18 (Latz et al., 2013; Gao et al., 2018). Therefore, NLRP3 inflammasome activation and release of mature IL-1β played a key role in CFA-induced pain and inflammation pathogenesis. Recent studies have also found that itaconate could dissociate NLRP3 from NEK7 by alkylation to inhibit the formation of inflammasome complexes and reduce the inflammatory progression of crystal-stimulated peritoneal inflammatory (Hooftman et al., 2020). In view of the fact that itaconate can inhibit NLRP3, and in our experiments, it was also found that DI reversed the activation of NLRP3 complex and the release of IL-1β caused by CFA. We speculated that the analgesic effects of DI might be related to the inhibition of NLRP3. In previous studies, it was also found that itaconate could play an anti-inflammatory role by promoting Nrf2. Whether Nrf2 is involved in the development of inflammatory pain still needs to be further explored. In the future, the mechanism of whether itaconate has other effects on pain relief still needs in-depth research. In this study, we concluded that the NLRP3 inflammasome complex was activated in the CFA-induced inflammatory pain model, which was consistent with the findings of Gao et al. (2018), and DI significantly reduced the expression of NLRP3, caspase-1, and IL-1β level in CFA-induced inflammatory tissues. These results indicated that DI may alleviate CFA-induced inflammatory pain by inhibiting the activation of the NLRP3 inflammasome as well as mature IL-1β.

## Conclusion

In this study, our experiments demonstrated that DI could alleviate the pain-like behavior of CFA mice by inhibiting the infiltration of plantar inflammatory cells and macrophage activation in DRG and microglia in the spinal cord. The analgesic behavior of DI was related to the inhibition of NLRP3 inflammasome. DI is expected to be a prospective candidate for the treatment of inflammatory pain management.

## Data Availability

The raw data supporting the conclusions of this article will be made available by the authors, without undue reservation.

## Abbreviations

Arg-1, Arginase-1; ASC, Apoptosis-associated speck-like protein; CFA, Complete Freund’s adjuvant; CNS, Central nervous system; DI Dimethyl itaconate; DRG, Dorsal root ganglion; IL-1β, Interleukin-1 beta; iNOS, Inducible nitric oxide synthase; NLRP3, Nod-like receptor protein 3; NF-κB, Nuclear factor kappa-B; PNS, Peripheral nervous system; TNF-α, Tumor necrosis factor-alpha.
